# A Comparative Assessment of Measures of Leaf Nitrogen in Rice Using Two Leaf-Clip Meters

**DOI:** 10.3390/s20010175

**Published:** 2019-12-27

**Authors:** Ke Zhang, Xiaojun Liu, Yong Ma, Rui Zhang, Qiang Cao, Yan Zhu, Weixing Cao, Yongchao Tian

**Affiliations:** 1 National Engineering and Technology Center for Information Agriculture, Nanjing Agricultural University, Nanjing 210095, China; 2017201080@njau.edu.cn (K.Z.); liuxj@njau.edu.cn (X.L.); 2019101044@njau.edu.cn (Y.M.); 2019101034@njau.edu.cn (R.Z.); qiangcao@njau.edu.cn (Q.C.); yanzhu@njau.edu.cn (Y.Z.); caow@njau.edu.cn (W.C.); 2 Key Laboratory for Crop System Analysis and Decision Making, Ministry of Agriculture and Rural Affairs, Nanjing Agricultural University, Nanjing 210095, China; 3 Jiangsu Key Laboratory for Information Agriculture, Nanjing Agricultural University, Nanjing 210095, China; 4 Jiangsu Collaborative Innovation Center for Modern Crop Production, Nanjing Agricultural University, Nanjing 210095, China; 5 College of Agriculture, Nanjing Agricultural University, Nanjing 210095, China

**Keywords:** flavonoid, nitrogen nutrient, nitrogen regulation, grain yield, nitrogen balance index

## Abstract

Accurate estimation and monitoring of crop nitrogen can assist in timely diagnosis and facilitate necessary technical support for fertilizer management. Four experiments, involving three cultivars and six nitrogen (N) treatments, were conducted in southeast China to compare the two leaf-clip meters (Dualex 4 Scientific+, Force-A, Orasy, France; Soil and Plant Analyzer Development (SPAD) meter, Minolta Camera Co., Osaka, Japan) for their ability to measure nitrogen nutrient-related indicators. The results indicated that Chl had a better monitoring accuracy for chlorophyll in per unit leaf area as compared to SPAD value, and there was no saturation to appear under high leaf chlorophyll concentration status. Flavonoids (Flav) presented the advantage of early diagnosis of rice N nutrition status (about one day as compared to SPAD value). As a reliable N nutrient diagnosis indicator, it also improved the estimation accuracy compared with the classical SPAD-based method. The other Dualex value also obtained good monitoring results. Flav was positively correlated with N deficiency, and with higher R^2^ in panicle initiation and booting stages with low RMSE, respectively; whereas SPAD value was negatively correlated with nitrogen deficiency. Therefore, the Flav-based nitrogen application model was found to provide an early rice nitrogen fertilizer application approach, especially in the panicle initiation and booting stages.

## 1. Introduction

Nitrogen (N) is an essential nutrient in agricultural crops, which is necessary to improve grain yield and quality [1]. While fertilizer overuse has led to several environmental problems, such as pollution from run-off, leaching, release into the environment, water eutrophication, and soil consolidation [1], its importance to agricultural productivity also cannot be underestimated. Rockström et al. [2] indicated that the global nitrogen circulation flux was beyond acceptable limits, and a large part of it came from the consumption of agricultural produce. Therefore, precision N application in crops has always been a primary focus of agricultural scientists. 

Efficient fertilizer management depends upon real-time and accurate evaluation and diagnosis of the nutritional status of crops [3]. Monitoring of crop growth and N status based on large-scale aerial and satellite remote sensing technology [4] has greatly contributed to the large-scale management of farmland. However, such expensive commercial methods are unsuitable for the needs of small farmers in southern China. Near-ground sensing technology is ideally suited to scientific management of crops growing in smaller fields because of their high portability, adaptability, and low cost [5]. It has two major components: canopy sensing technology and blade horizontal sensing technology. Canopy sensing technology uses a non-contact remote sensing method to monitor the canopy reflectance of a specific area, and then extrapolate the nitrogen nutrition status of crops [6]. This method is mainly constrained by the structure of crop canopy and its biochemical components. The sensitivity of chlorophyll concentration in the canopy under different nitrogen treatments is low [7]. In 2012, Foster et al. [8] used a three-band index (transform chlorophyll absorption ratio index, TCARI) to predict the chlorophyll content of switch-grass leaves. However, if the chlorophyll content was lower than 10 µg cm^−2^, there was a positive correlation between TCARI and chlorophyll content but a negative correlation between TCARI and chlorophyll content when chlorophyll content was at 10–70 µg cm^−2^. 

At the leaf level, Sims and Gamon [9] used data collected by FieldSpec sensor (Analytical spectral devices, Ins., Boulder, CO, USA) and LI-1800 (LI-COR Inc., Lincoln, NE, USA) to calculate the mSR705 difference between the top leaf (LFT1) and the third leaf of rice (LFT3) (mSR705 L1-mSR705 L3). They found that the difference could be used to monitor the N nutrient status efficiently when the N fertilizer was abundant [10]. They found that when the blue band reflectance (λ = 445 nm) was added to eliminate the reflection of light from the blade surface, the sensitivity of detection was enhanced to small differences in chlorophyll concentration. Wang et al. [11,12] indicated that the difference in SPAD value (SPAD meter, Minolta Camera Co., Osaka, Japan) between LFT4 and LFT3 could be used to monitor rice N nutrient status. In recent years, studies have proposed a variety of indices such as, difference SPAD index (DSI, SPAD_L1_-SPAD_L3_) [13], ratio SPAD index (RSI, SPAD_L1_/SPAD_L3_) [14], normalized SPAD difference index (NDSI, (SPAD_L1_-SPAD_L3_)/(SPAD_L1_-SPAD_L3_)) [13], relative SPAD difference index (RDSI, (SPAD_L1_-SPAD_L3_)/SPAD_L3_) [11], and difference position index (DPI, (SPAD_Li_-SPAD_L4_)/SPAD_L4_, i = 1, 2, 3 [12], normalized SPAD index (NSI, SPAD_Li_/well fertilizer-SPAD_Li_, i = 1, 2, 3, 4) [15].

Muñoz-Huerta et al. [6] found that the SPAD value prompted a saturation phenomenon once the amount of N fertilizer was abundant. It implied that when using the SPAD value under high N conditions, it would be difficult to distinguish the difference among N nutrition status across various plots. In 2007, Wu et al. [16] reported that the SPAD value lagged behind variations in N levels during crop N monitoring. For example, SPAD value decreased or increased after a change in petiole nitrate concentration. Chen et al. [17] found that when nitrate-N was the only N source, the increase of rhizosphere pH led to the slow growth and yellowing of new leaves, which was mainly attributed to an increase of phenolic compounds in the phenylpropane metabolism pathway in roots under high pH conditions. Meanwhile, Schijlen et al. [18] also found that more rutin and other flavonoid polyphenols appeared when *Arabidopsis thaliana* seedlings grew for two days under N deficiency conditions. In 2010, Cui and Paek [19] studied the effects of different ammonium-nitrate ratios on secondary metabolites in adventitious root culture of *Hypericum*, and reported that the optimum level of total phenols and flavonoids was produced when the ammonium-nitrate ratio of the medium was 5:25. Strissel et al. [20] reported that the activity of phenylalanine ammonia-lyase led to a decrease in the total content of flavonoids in high N treatments. Therefore, the concentration of polyphenols in plants (flavonoids, anthocyanins, etc.) could be used to monitor the N nutrition status in discrete N conditions, both directly and rapidly.

Plant polyphenols are secondary metabolites of carbon-based polyphenols, flavones, and their derivatives. As a group of polyphenols, they accumulate more in monocotyledonous plants such as rice (*Oryza sativa* L.), maize (*Zea mays* L.), and wheat (*Triticum aestivum* L.) [21]. Following the analysis of flavonoids by LC-ESI-MS/MS-based targeted metabolism, Dong et al. [22] found flavonoid accumulation to be higher in flag leaves than in other organs (stem, panicle, and grain). Therefore, plant leaves could be used for monitoring the concentration of flavonoids.

Dualex 4 Scientific (Force-A, Orasy, France) stimulates chlorophyll fluorescence of leaf epidermis by ultraviolet radiation (375 nm) to estimate flavonoid content in the leaves of crops, and uses it to estimate the N nutrition status of crops [23]. Zebarth et al. [24] proved that the accuracy of crop N status diagnosis increased when the UV-based Dualex meter was used [25]. In recent years, research on plant N nutrition diagnosis based on flavonoids has focused on dryland crops such as wheat [26], maize [27], or horticultural crops such as grapes and apple [20]. The preliminary results proved that flavonoids played an important role in nitrogen diagnosis in different crops. Jose et al. [28] reported that Flav could accurately measure maize N status as compared to SPAD value. Franciso et al. [29] also demonstrated that Dualex meter (a small, light, hand-held, leaf-clip meter) flavanols measurement on both sides of the leaf is straightforward and feasible for an on-farm measurement. Therefore, more attention should be accorded to the diagnostic characteristics and measurement of nitrogen nutrition in annual aquatic herb rice in future research.

The main objectives of this study are: (1) to test the ability of Dualex to estimate flavonoids and chlorophyll in rice leaves; (2) to evaluate the diagnostic effect of Dualex and SPAD on nitrogen nutrition in rice; and (3) to establish a diagnostic method of nitrogen nutrition in rice based on flavonoids. The results will potentially provide a justification for precise nitrogen management in agricultural crops based on the Dualex instrument.

## 2. Materials and Methods

### 2.1. Experimental Design

Japonica rice is the main rice variety type in Jiangsu, China. Jiangsu also has the highest average rice grain yield in China [30,31]. The present study was carried out in two typical japonica rice-producing areas in the Jiangsu Province, including the lower reaches of the Yangtze River (Rugao) and Huaihe River Basin (Huai’an and Sihong) in Northern Jiangsu. The experiment factors included different years, diverse varieties of japonica rice, and multiple nitrogen levels. In Huai’an and Sihong, the base soil strength was lower than that in Rugao, with the same nitrogen gradient appropriate to increase the amount levels of nitrogen. The basic information and details of the location, as well as growing conditions, are as follows (Table 1).

Field experiment 1: The experiment was conducted over a single season in 2016 from June to October in Rugao (Experiment 4). Wuyunjing-24 and Ningjing-4 were sown on May 15 and transplanted on 15 June with a density of 15 cm × 30 cm. The total plot area was 35 m^2^ (7 m × 5 m). Four N fertilization rates 0 (N0), 100 (N1), 250 (N4), and 400 (N6) kg∙ha^−1^ were applied in the form of urea at a rate of 30% at pre-planting, 30% at tillering, 20% at jointing, and 20% at the booting stage. In addition, a treatment of topdressing lacking (T0) was set in each nitrogen gradient. Supplemental applications of 127 kg∙ha^−1^ P_2_O_5_ and 225 kg∙ha^−1^ K_2_O as potash fertilizer were also applied to all the experimental plots.

Field experiment 2: The experiment was conducted over a single season from June to October of 2015 in the Sihong (Experiment.1 and 3) and Huai’an (Experiment.2) regions of China. Varieties of Wuyunjing-24, Ningjing-4, and Lianjing-7 were sown on 20 May and transplanted on 20 June. The experiment had four N fertilization rates of 0 (N0), 120(N2), 240 (N3), and 360 (N5) kg∙ha^−1^, respectively. The other factors and growing conditions were identical as Experiment 1. Details of the experiments are presented in Table 1.

### 2.2. Agronomical Data Acquisition and Determination

#### 2.2.1. Sampling Period and N Concentration Determination

From the tillering (TI) to grain filling stages (GF), five randomly selected hills from each plot were sampled for growth analysis. Fresh leaves were collected and oven-dried at 80 °C for 48 h. Leaf dry matter was measured using this material, followed by determination of the leaf N concentration (LNC) using the micro-Kjeldahl method [32].

#### 2.2.2. SPAD and Dualex Values Measurements

Dualex (Dualex Scientific+, Force-A, Orasy, France) and SPAD (Minolta Camera Co., Osaka, Japan) values of the four fully expanded uppermost leaves were determined from TI to GF growth stages. The first, second, third, and fourth fully expanded leaves from the top of the plant were designated as a leaf from top 1, 2, 3, and 4, respectively, as counted from the top. Dualex and SPAD readings were taken at three locations: 1/3 (a), 1/2 (b), and 2/3 (c) of the distance from the leaf base, respectively. Means of the distances were combined and recorded. The mean of (a,b), (a,c), (b,c), and (a,b,c) corresponded to (a), (b), and (c) positions, respectively. Ten randomly selected plants from each plot were measured in the field. For the sake of illustration, Sa, Sb, and Sc represent Dualex and SPAD readings at leaf locations (a), (b), and(c), respectively. Sabc represents the average Dualex and SPAD readings of the whole leaf [33]. In this study, the Dualex meter and SPAD meter were used to monitor the leaf N status before and after topdressing. The leaf N status was measured at one day before spikelet-promotion, at fertilizer topdressing, on the day of topdressing, one day after topdressing, and two days after topdressing, respectively.

#### 2.2.3. Chlorophyll (Chl) and Flavonoid (Flav) Content Determination

After following scanning, leaf samples were cut into fine pieces (<0.25 mm^2^) and chlorophyll was extracted using 96% (*v*/*v*) ethanol. Solutions were stored for up to 24 h in darkness (to prevent chlorophyll degradation) until all chlorophyll was extracted, as indicated by white leaf tissue. Chl contents were then determined by measuring absorbance at 652 and 665.2 nm wavelengths on a UV–Vis spectrophotometer; Chl concentration (µg cm^−2^) was calculated as given in Equation (1) [34].

(1) $${\mathrm{Chl}}_{\left(a+b\right)}=22.12\times A_{652.0}+2.71\times A_{665.2}$$

Flav content determination was conducted as follows: (1) 0.2 g of a leaf was dried at 80 °C in a forced draft oven until it reached a constant weight. It was then transferred to a 10 mL volumetric flask and extracted with 70% ethanol in a constant volume for 10 h; (2) taking 1 mL of extraction solution from Step 1, 0.5 mL of 5% NaNO_2_ was added and mixed by gentle shaking for 5 mins; (3) to the mixed solution from Step 2, 0.5 mL of Al(NO_3_)_3_ was poured into the mixed solution. The solution was allowed to stand for 6 mins; (4) 2 mL of NaOH was added to the previous mixture of liquids and allowed to stand for 10 mins. The content of flavonoids in rutin was determined by a UV-2400 spectrophotometer at 510 nm [35].

#### 2.2.4. Nitrogen Nutrition Index (NNI)

The Nc curve model used in this work was reported by Ata-Ul-Karim et al. in 2013 [36] (Equation (2)). The NNI was calculated based on Equation (3):

(2) $$N_{c}=3.53\times W^{-0.28}\;\left(W\geq 1.55{\;t\;\mathrm{ha}}^{-1},{\;R}^{2}\;=\;0.80\right)$$

(3) $$NNI=\frac{N_{a}}{N_{c}}$$

where *N_c_* (%) is the N concentration critical value, *W* is the dry weight of plant (t ha^−1^), and *N_a_* (%) is the measured N concentration of crops.

#### 2.2.5. Nitrogen Sufficiency Index (NSI)

Equation (4) was used to calculate NSI [5]:

(4) $$NSI=\frac{N_{TP}}{N_{WFTP}}$$

where *N_TP_* (%) is the N concentration of measured plot and *N_WFTP_* (%) is the N concentration of sufficiency plot [5].

#### 2.2.6. Accumulated Nitrogen Deficit (*AND*)

Accumulated N deficit (kg N ha^−1^) at different growth stages can be was calculated using Equation (5) [37]:

(5) $$AND=N_{cna}-N_{na}$$

where *N_cna_* represents N accumulation under critical N growth conditions and *N_na_* represents N accumulation under different N levels. When AND = 0, plant N nutrition is optimal. When AND > 0, plant N nutrition status is deficient. When AND < 0, the plant has excessive N nutrition.

#### 2.2.7. Calculation of Relative Accumulated Growing Degree Days (*RAGDD*)

In this paper, the Relative Accumulated Growing Degree Days (RAGDD) was used as the time variable in a dynamic change model. *RAGDD* was calculated from *AGDD* (Accumulated Growing Degree Days), which is the summation of GDD (Growing Degree Days) throughout the experiment [38]. Meteorological data including temperature were collected using the automated weather station Dynameta-1K (Dynamax Inc., Houston, TX, USA) installed at each test site and recorded every 5 min using the EM50 data acquisition system (Decagon Devices Inc., Washington, DC, USA). *AGDD* was calculated as follows:

(6) $$AGDD=\overset{n}{\underset{i=1}{\sum }}\left(\frac{T_{2:00}+T_{8:00}+T_{14:00}+T_{20:00}}{4}-T_{BASE}\right)$$

where *T*_2:00_, *T*_8:00_, *T*_14:00_, and *T*_20:00_ are the temperature at 2:00, 8:00, 14:00, and 20:00 of the day. *T_BASE_* is the base temperature, which is usually set to 12.5 °C for Japonica rice [39]:

(7) $$RAGDD=\frac{AGDD_{i}}{AGDD_{harvest}}$$

where *AGDD_i_* is the *AGDD* of the day and *AGDD_harvest_* is the *AGDD* of harvest, the accumulated growing degree days of the whole entire growth period.

#### 2.2.8. Statistical Analysis 

This study used GraphPad Prism 5 (GraphPad Prism 5, GraphPad Software, San Diego, CA, USA) for data analysis and the creation of figures. The data from Experiments 1, 3, and 4 were used for the model to develop the model for meters’ values and for N indicators in the different various growth periods. The data from Experiment 2 was used for the test model. Coefficient of determination (R^2^), root mean square error (RMSE), and relative RMSE (RRMSE) were used to assess the stability of the models developed in this study:

(8) $$\mathrm{RMSE}\;=\sqrt{\frac{1}{n}\times \sum_{i=1}^{n}{\left(P_{i}-O_{i}\right)}^{2}}$$

(9) $$\mathrm{RRMSE}\;=\;\mathrm{RMSE}\;/\bar{{\;O}_{i}}\times 100\%$$

where n is the number of test samples, Pi is the model estimate, Q_i_ is the observed value, and $\bar{{\;O}_{i}}$ is the average observed value [39,40].

For each sampling date, year and cultivar of winter wheat cultivar, the amounts of PDM, LDM, SDM, and LAI produced with the various N rates. The corresponding tissue N concentrations were subjected to analysis of variance (ANOVA) using general linear model (GLM) and ANCOVA test procedures in IBM SPSS Version 20.0 (IBM Corporation, Armonk, NY, USA). Each critical N concentration was defined by the point at which the parameter of the dilution curve was markedly inflected sharply. A multiple comparisons test was used (Least-Significant Difference, LSD; *p* < 0.05) to detect significant pairwise treatment effects.

## 3. Results

### 3.1. Relationship between Leaf-Clip Values (Flav, Chl, and SPAD) and Measured Values in Leaves

**Figure 1:**
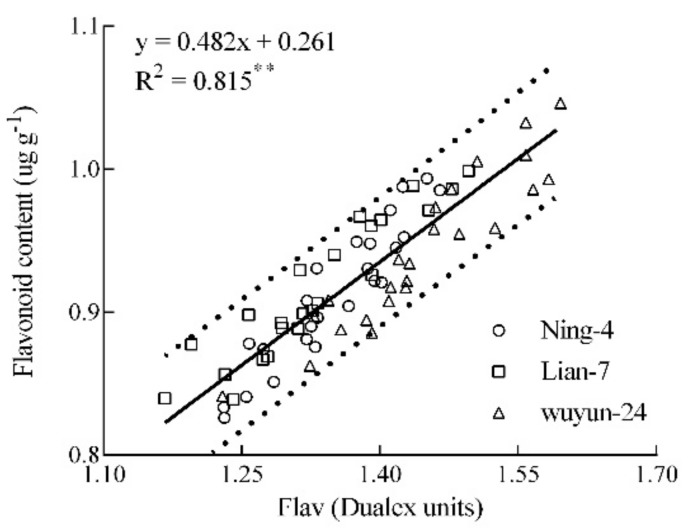
The relationship between leaf flavonoids content and Dualex values.

**Figure 2:**
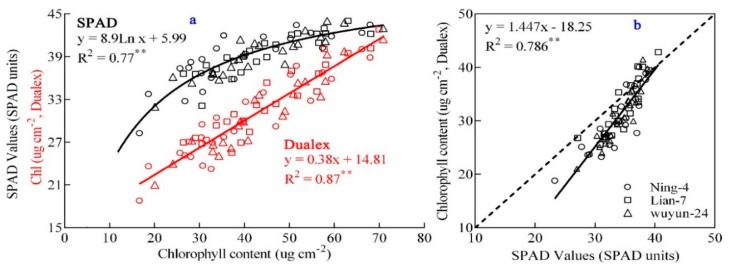
Scatterplot between SPAD values and leaf chlorophyll or Chl values (Dualex meter’s values). (**a**) Represents the relationships between meter’s values and chlorophyll content; (**b**) the relationship between SPAD value and Dualex value.

Figure 1 shows a positive relationship between measured Flav and meter’s Flav in the various growth stages across varieties (R^2^ = 0.82, *p* < 0.01). The range of fresh leaf Flav was 1.20–1.57 for the Dualex unit, while the measured flavonoid ranged from 0.83 to 1.07 µg·g^−2^ (Figure 2). In summary, the Dualex meter was an effective index to estimate rice leaf flavonoid values.

A strong correlation was also observed between the meters’ values (Chl, Dualex meter calculated value; SPAD, and SPAD meter) and measured chlorophyll value (R^2^ = 0.87 and 0.77, *p* < 0.01, Figure 2a). A linear regression plot was generated between the Chl value and the measured chlorophyll value (the range of Chl was from 18.9 to 42.9 µg·cm^−2^, Figure 2a), while the power function model was used to show the correlation between the SPAD value and the measured chlorophyll value (28.3 > SPAD > 45.6, Figure 1). The Chl value could also be used to calculate the leaf SPAD value (R^2^ = 0.786, *p* < 0.01, Figure 2b) and a higher R^2^ observed for leaf chlorophyll content (Chl value: R^2^ = 0.87, SPAD value: R^2^ = 0.77, *p* < 0.01, Figure 2a) could be attributed to chlorophyll saturation leading to a decreased accuracy of monitoring. Therefore, Chl was a more effective indicator to monitor real-time leaf chlorophyll content.

### 3.2. Sensitivity Analysis of Dualex Meter and SPAD Meter in N Nutrition Status 

**Figure 3:**
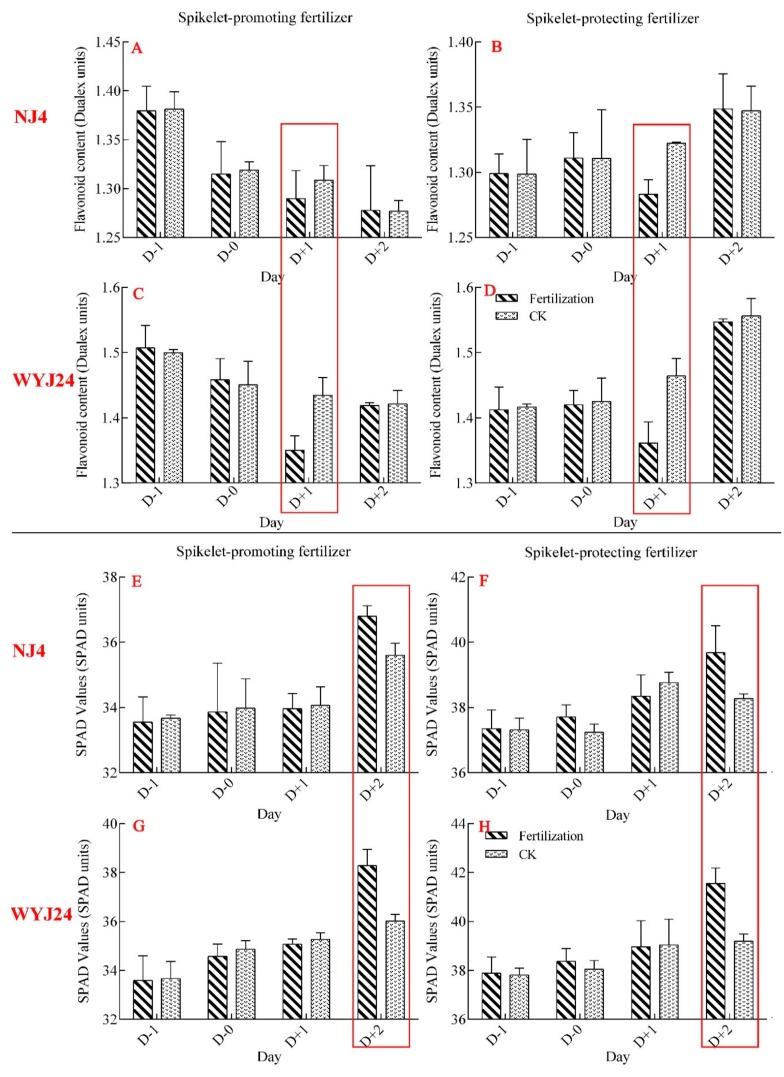
Variation of flavonoid (Flav) value or SPAD value between before and after fertilization under different varieties. (**A**–**D**) Shows the changes of Flav value in two varieties; (**E**–**H**) represents the trends of SPAD values before and after fertilization.

For the two varieties (Ningjing-4 and Wuyunjing-24), the Flav value decreased at one day after topdressing (Figure 3A,C) while the SPAD value increased two days after topdressing (Figure 3E,G). This study also explored the trends of Flav and SPAD value during spikelet-protecting fertilizer topdressing. The Flav value of the fertilizer application plot was significantly lower than the unfertilized treatment (Figure 3B,D) and the SPAD value of fertilizer application plot was significantly higher than the unfertilized treatment (Figure 3F,H). These results indicated that Flav responded more rapidly to nitrogen status in rice than SPAD, implying that SPAD was less effective in monitoring the changes of nitrogen content in rice.

### 3.3. Dynamic Characteristics of Dualex and SPAD Values Under Different Treatments

**Figure 4:**
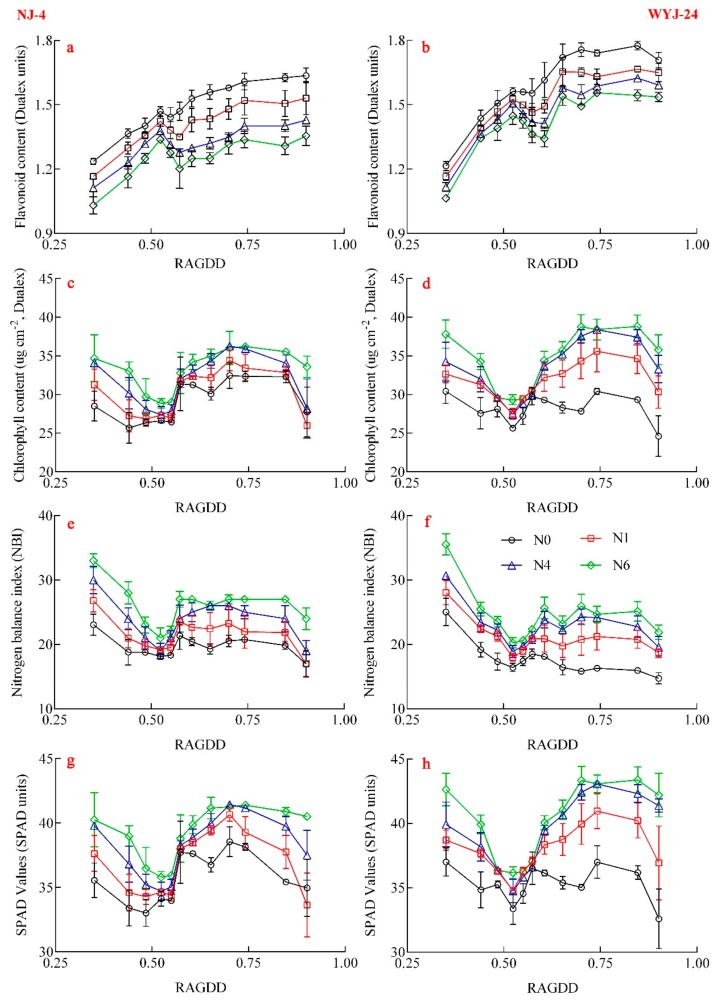
Changes of Dualex values. (**a**,**b**) Flav; (**c**,**d**) Chl; (**e**,**f**) NBI; (**g**,**h**) SPAD values under different N fertilizer rates. (**a**,**c**,**e**,**g**) Trends of NJ4 (Ningjing-4); (**b**,**d**,**f**,**h**) represent the changes of WYJ24 (Wuyunjing-24).

The dataset from Experiment 3 was used to analyze the dynamic changes of Dualex value and SPAD value in the total growth stages (Figure 4). The results indicated that the trends of all meters’ indicators were similar for both cultivars, while the difference among N treatments was more diverse.

For Flav values, the same dynamic changes appeared in two different varieties and produced a bell-shaped model until the early stem elongation (SE) stage, which subsequently decreased until the panicle initiation stage and increased again until chlorophyll saturation. The Flav value ranged from 1.03 to 1.63 in the Ningjing-4 cultivar whereas, in the Wuyunjing-24, cultivar, the values ranged from 1.06 to 1.77. The trends for Chl and SPAD values were the opposite of those observed for Flav values, where the NBI decreased before the SE stage, and then increased to saturation, before declining again. 

Following the analysis of the Flav trends of different cultivars, it was observed that Flav value could be used to distinguish between the different N rates (the order of Flav is N0 > N1 > N2 > N3, Figure 4a,b). For other meters’ index, Chl and SPAD values could only distinguish among the N0, N1, and N2 treatments in most growth stages. With N3 and N2 treatments, the leaf color turned yellow from later-tillering stage to the early-SE stage (0.5 < RAGDD < 0.65) due to pathophysiology. As a result, the chlorophyll-related indices (Chl and SPAD) could not be used to differentiate between N2 and N3 when the N status was low (Figure 4b,c,g,h). NBI is given by the ratio of Chl and Flav, which also was unable to detect the difference among the diverse N levels.

**Figure 5:**
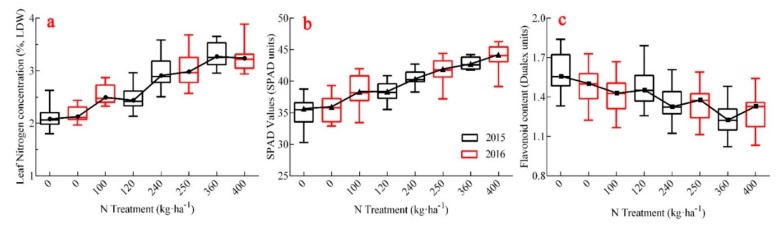
Trends of leaf nitrogen content, SPAD value, and Flav value under different N fertilizer rates. (**a**) Trend of leaf nitrogen concentration; (**b**) represents the changes of SPAD value; (**c**) shows the trends of flavonoid content.

The study also generated a boxplot to describe the characters of leaf N concentration (LNC), Flav, and SPAD values in different N treatments. Figure 5a showed that LNC continued to rise with an increase in N and produced an ‘S’-shaped curve, with a median biased toward the smaller sides, indicating left skewness for LNC distribution (Figure 5a). The Flav value decreased as the N rates increased, which was the opposite of the change in LNC; the distance between the upper quartile and the lower quartile of the box plot was far and relatively uniform. It indicated that the Flav value was an appropriate diagnostic index for the nitrogen level, which could differentiate the nitrogen levels of different varieties across growing periods (Figure 5c). The SPAD value increased slowly with an increase in the N application rate, but it could not accurately estimate leaf nitrogen content especially in high nitrogen areas (400 kg ha^−1^). In addition, the median SPAD value deviated from the central position, while the nitrogen level was widely distributed in the larger or smaller value areas. When LNC was larger than 2.89% (N treatment = 240 kg ha^−1^), the distance from the upper quartile to the lower quartile was smaller. One step indicated that the SPAD value decreased under conditions of excessive nitrogen and was not effective in differentiating different cultivars and growth stages as a result of chlorophyll saturation (Figure 5b).

### 3.4. Relationship between N Indicators and Meters’ Index (Dualex Meter: Flav, Chl, and NBI; SPAD Meter: SPAD Value)

There was a positive correlation between Chl, NBI, SPAD values, and N indicators, while a negative correlation was observed between Flav values and N indicators. Analysis was carried out based on Experiment 1, 3, and 4 test data, Dualex values (Flav, Chl, NBI), SPAD values, and determination coefficients (R^2^, Table 2) of nitrogen nutrition indices (LNC, PNC, NNI, NSI) from tillering to maturity in different years (R^2^, Table 2). The results showed that there were significant correlations between the different indicators and their spectrographic values, indicating that the spectrographic values could effectively monitor the nitrogen nutritional status of rice. The determination coefficient R^2^ ranged from 0.34 to 0.84 (*p* < 0.05). However, the correlation was different at different growth stages. 

The lower determinant coefficients mainly occurred at the tillering stage, jointing stage, and flowering stage. The higher determinant coefficient R^2^ was observed from the jointing stage to the booting stage, with R^2^ ranging from 0.61 to 0.84 (*p* < 0.01). The determinant coefficient R^2^ between Flav values and N indicators in each corresponding growth stages was higher (0–0.17) than that between SPAD values and N indicators; the determination coefficient R^2^ for Chl, NBI, values, and N indicators was also lower than R^2^ between Flav values and N indicators. It was inferred that Flav value could be more effective than chlorophyll value to estimate N deficiency in rice leaves. The determinant coefficient R^2^ between NNI and different spectrographic values (0.56–0.84, *p* < 0.01) was smaller than other N indicators, but the correlation coefficient was higher (LNC: 0.35–0.83, PNC: 0.43–0.76, NSI: 0.34–0.84, *p* < 0.01). This indicated that the monitoring accuracy was obviously superior to other indicators, especially in the key diagnostic and regulation stages of nitrogen fertilizer (jointing stage to booting stage). In the analysis of N nutrition status during the whole growth stages, it was found that the inversion accuracy of Flav value for all N indicators exceeded 69%, and the accuracy of indicators such as SPAD value was slightly lower than Flav value (SPAD: 60%–70%, Chl: 52%–73%, NBI: 68%–75%).

For further understanding of the determination of N deficiency in rice, this study provided an in-depth analysis of the relationship between spectrographic indicators and N deficit and grain yields (Table 2). The results showed that spectrographic value can effectively predict accumulated N deficiency (AND) and grain yield at different growth stages, which implied that the accuracy of predicting accumulated N deficient reached more than 55% (R^2^ > 0.55, *p* < 0.01), while the accuracy of predicting grain yield was slightly lower (0.32 < R^2^ < 0.64, *p* < 0.05). Further analysis in different growth stages revealed that while the monitoring effect of N deficiency was better from the tillering stage to the panicle initiation stage and the flowering stage (R^2^ > 0.63, *p* < 0.01), it was not ideal or slightly decreased in the middle or late growth stages (R^2^ > 0.61, *p* < 0.01). In yield prediction studies, the effect of early growth stages (tillering stage, jointing stage) and late growth stages (heading stage, flowering stage) was better than the middle growth stages (panicle initiation stage and booting stage).

### 3.5. The Threshold of Conventional Japonica Rice N Application Based on Flav Value

**Figure 6:**
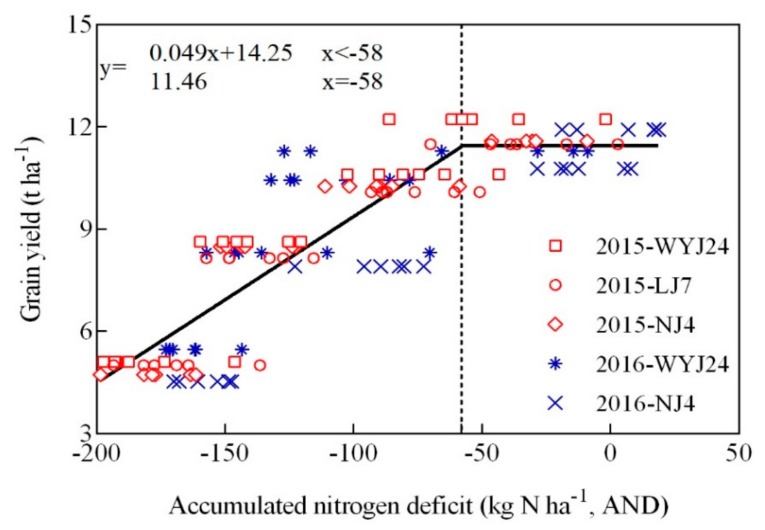
Relationships between accumulated nitrogen deficit (AND) and grain yield during vegetative growth period under varied N rates.

**Figure 7:**
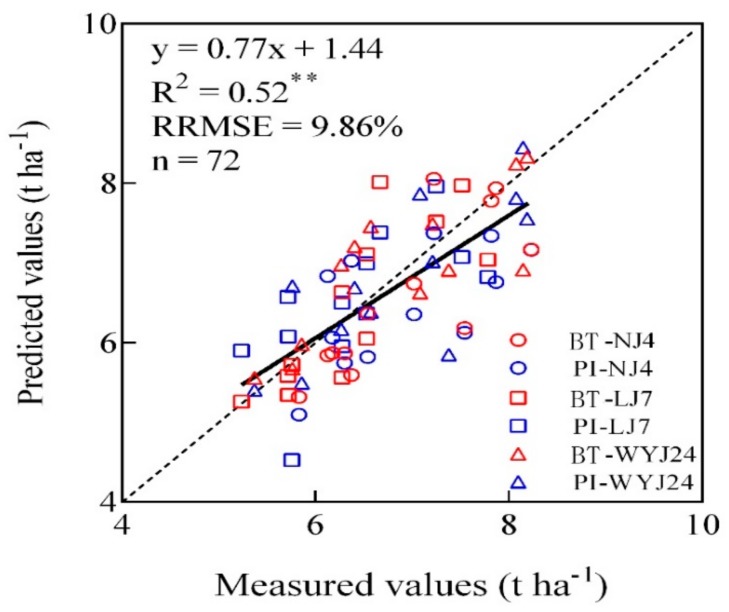
Relationships between measured and predicted grain yield values of three Japonica rice (NJ-4, Ningjing-4; LJ7, Lianjing-7; WYJ24, Wunyunjing-24) at booting (BT) and panicle initiation (PI) stages.

**Figure 8:**
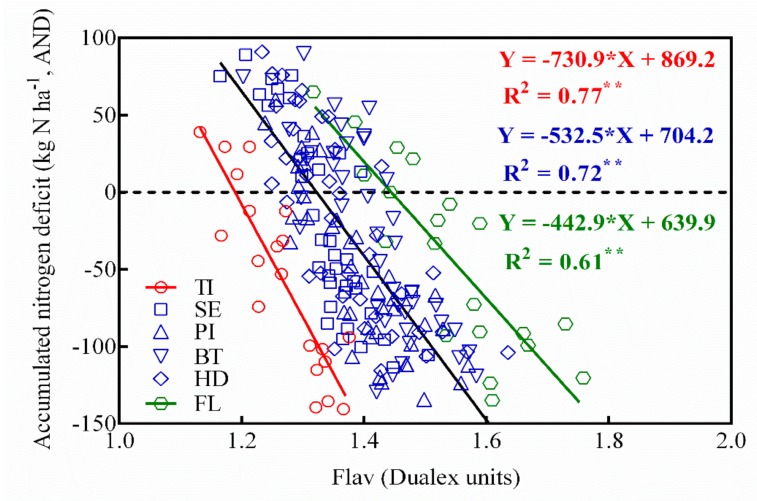
Relationships between Flav values and accumulated nitrogen deficit (AND) during vegetative growth period.

**Figure 9:**
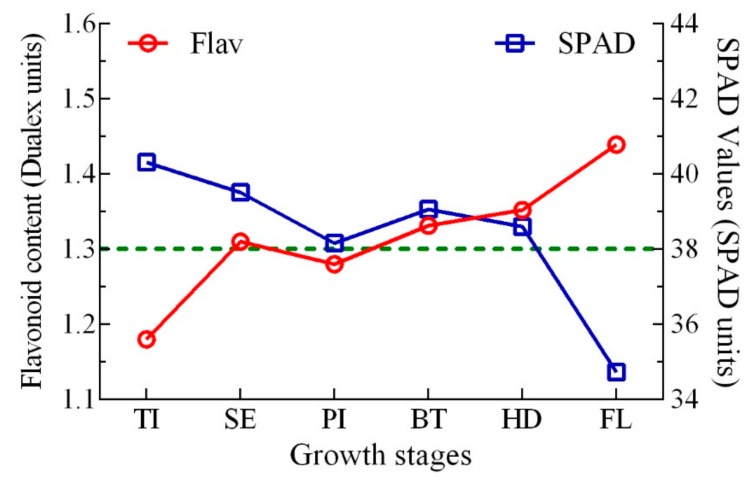
Time series changes of thresholds Flav values (TI: tillering, SE: stem elongation, PI: panicle initiation, BT: booting, HD: heading, FL: flowering).

This work studied the relationship between accumulated nitrogen deficit (AND) and grain yield based on Experiments 1, 3, and 4 test data (Figure 6). The results showed that there was a positive correlation between them and AND could explain 68% of the variability of crop yield with a relative root mean square difference (RRMSE) of 9.86% (Figure 7). The predicted model is a linear plus platform structure equation, and the threshold was set at AND = 0 kg N ha^−1^. When AND is less than 0 kg N ha^−1^, the predicted equation changes linearly, showing an N deficit; when AND is more than 0 kg N ha^−1^, the yield is basically unchanged, indicating that N is sufficient. At the same time, the analysis of results in Table 2 revealed that the Flav value had a good monitor effect on N indicators and grain yield, and the inversion accuracy (R^2^) of each indicator was higher than that in other instrument-based monitoring indicators. Figure 8 established a regression relationship between Flav value and accumulated nitrogen deficit (AND). It is evident from the figure that the monitoring accuracy was higher at the tillering stage and flowering stage (R^2^ > 0.72, *p* < 0.01), whereas the inversion accuracy from the jointing stage to the heading stage (R^2^) was higher than 0.6. Therefore, the relationship between Flav value from the jointing stage to the heading stage and AND was established (R^2^ = 0.61, *p* < 0.01). Combined with platform value when AND = 0 kg N ha^−1^ in Figure 6, the change trend chart of threshold (Figure 9) was obtained. As shown in the figure, the threshold of the tillering stage and flowering stages is clearly different from each other, while the threshold from the stem elongation stage to the heading stage fluctuated around 1.3. It implies that the threshold of Flav value is 1.3 Dualex unit and the threshold of SPAD value is 38 SPAD unit in the critical fertilization stage. When the measured Flav value was higher than 1.3 Dualex unit or when the measured SPAD value was lower than 38 SPAD unit, the additional N fertilizer needed was calculated as presented in Figure 6. On the contrary, when the measured Flav value was lower than 1.3, and the Dualex unit or measured SPAD value was higher than 38 SPAD unit, it could be concluded that the rice crop was in a nitrogen sufficient state and N fertilizer topdressing were not needed.

## 4. Discussion

### 4.1. Chlorophyll Estimation Based on Dualex and SPAD 

In 1963, the leaf-clip-type sensors to estimate Chl content in leaves based on apparent leaf transmittance were first used to monitor Chl content in living rice leaves [41]. The SPAD meter (SPAD instrument), developed by the Ministry of Agriculture, Forestry and Fisheries of Japan has found relatively wide use as a single-leaf spectrometer to test the value as a dimensionless relative value, but it is greatly influenced by leaf structure [42]. The Dualex 4 Scientific is a reliable leaf fluorescence sensor that can estimate Chl content and epidermal polyphenol content. Its biggest advantage is the ability to use chlorophyll fluorescence (λ = 375 nm) to monitor flavonoid content and using the new red-edge band (λ = 710 nm) and the new near-infrared band (λ = 850 nm) to estimate Chl content [43].

This study showed that the Dualex had better chlorophyll estimation accuracy as compared to SPAD, and was not limited by the saturation phenomenon in conditions of high chlorophyll concentration. It is consistent with the results of Cerovic et al. [41] and Coste et al. [43]. These observed differences in the various estimation indices can be potentially attributed to factors such as band difference. SPAD uses a band of 650 nm red light, which is the absorption band of chlorophyll, but it will appear to have achieved a high chlorophyll concentration and lead to lower measurement accuracy [41]. The Dualex uses the red-edge band at 710 nm. The red-edge band can reduce the saturation phenomenon of Chl content in high concentrations, where the 710 nm band had a smaller ‘sieve effect’ (transmittance values larger than in a homogenous sample), which can also help avoid interference from the presence of anthocyanins [44]. Carter and Spiering [45] showed that the ratio of transmittance at 850 nm and 710 nm had the best correlation with Chl content in leaves. In addition, the measured area of SPAD was 6 mm^2^, while that in Dualex was 19.6 mm^2^. The smaller area measured in SPAD is susceptible to factors such as blade structure (such as vein), so the SPAD value fluctuates greatly [23]. Therefore, Dualex has more advantages than SPAD in the monitoring of chlorophyll and other nutrient parameters of crop leaves.

### 4.2. Discrimination and Sensitivity of Rice Nitrogen Levels by Dualex Meter and SPAD Meter

Flavonoids are ubiquitous plant secondary products, and the accumulation of flavonoids is regulated by the structural genes of encoding flavonoids. Nitrogen directly participates in or affects the synthesis and accumulation of flavonoids [21]. Dualex uses the spectral characteristics of leaf epidermis flavonoids in the UV-A region to estimate the nitrogen content [23], implying that Dualex could effectively monitor the content of flavonoids in plants. This study demonstrated that Dualex could serve as an effective index to estimate the content of flavonoids in rice leaves because of the high correlation coefficient between the Flav value measured by Dualex and the measured flavonoids value, which is consistent with previous reports [21]. Meanwhile, this study also showed that Flav value can effectively distinguish between different nitrogen levels from the tillering stage to the filling stage because Flav value decreases with the increase of nitrogen application. It also indicates that there is a negative correlation between Flav value with nitrogen level. The conclusion is consistent with similar reports from dryland wheat [26] and maize [27]. In addition, this study found that the change of Flav values of Ningjing-4 and Wuyunjing-24 occurred one day earlier than SPAD values subsequent to nitrogenous fertilizer application. The results showed that Flav value was more sensitive to the change of nitrogen level in rice and had the ability to diagnose early N nutrition changes in rice, which was consistent with the conclusion that NO^3−^ could significantly reduce the content of flavonoids in Arabidopsis [18]. However, more physiological experiments should be conducted to explore the change of leaf flavonoids in future studies.

### 4.3. The Threshold of Rice Nitrogen Nutrition Diagnostics Based on Dualex 

The monitoring and diagnosis of rice nitrogen nutrition has been a key consideration in the precise management of rice cultivation [3]. Compared with SPAD value, the Dualex value was more time-efficient and stable in monitoring crop nitrogen status. Flav value also had a better impact on monitoring and diagnosis of crop nitrogen nutrition as compared with Chl and NBI, and was less impacted by the growth stage. Additionally, it also had a robust ability to monitor diverse varieties. These results are consistent with the monitoring results in wheat as reported by Cartelat et al. [26] using Dualex. Studies on the nutrition and physiology of rice showed that the root system of rice had a weak absorption capacity, slow biomass accumulation, and a smaller demand for nitrogen at the early growth stage, and only 20–30% of nitrogen from commercial fertilizers was absorbed and utilized by the crop [46]. Therefore, applying a large amount of nitrogen fertilizer at the early growth stage of rice had little influence on the final yield, thereby reducing the utilization of nitrogen fertilizer and increasing the cost of production. In addition, there also have been studies that showed that the application of nitrogenous fertilizer at the heading stage caused crops to be green, mature later, and reduce the final yield [47]. Rene and Birte [48] demonstrated that rice at the heading stage could grow and mature normally just by absorbing soil nitrogen, which meant that no corresponding nitrogen tracing measures were needed after the heading stage in the late growth stage of rice. In conclusion, the middle growth stage (stem elongation stage, booting stage) of rice is the peak stage of nitrogen demand, during which fertilization can effectively improve the utilization rate of nutrients in the fertilizer. This paper analyzed the correlation between different N indicators and single-leaf spectral indices in diverse growth stages (SPAD value, Chl value, Flav value, and NBI value) and found that the sensitive stages of single-leaf spectrometer monitoring were from the jointing stage to the booting stage, which was consistent with previous studies [15]. At the same time, we also found that the monitoring effect of Flav value was better than others after an overall analysis of multiple factors. Nitrogen nutrition index (NNI) is an effective tool to diagnose N deficiency in crops but for the acquisition of NNI value it was necessary to destroy the sample, which resulted in poor timeliness [37,49]. This study shows that Flav value can estimate NNI effectively. Meanwhile, the accuracy of Flav value in predicting rice N deficiency from early panicle initiation stage to booting stage was better than at other stages. Low root mean square error indicated that Flav value could be used to diagnose N deficiency in rice.

This work established the linear plus plateau regression model of N deficiency and grain yield. The findings were consistent with the results of Ata-Ul-Karim et al. [37]. The model structure showed that 0 kg N ha^−1^ was the threshold of N application in rice, the threshold of Flav value was 1.28, and 1.33 Dualex units (Figure 9) corresponding to the N deficiency at the jointing stage and the booting stage, respectively, in the key growth stages of rice. Further analysis showed that the threshold of Flav value fluctuated around 1.3 Dualex units from the stem elongation stage to the booting stage. This period was also the optimal period for N diagnosis by the Flav approach [15]. The most optimal threshold of SPAD value was at 38 SPAD units, which was consistent with the conclusion of Peng et al. [50]. Therefore, as a result of the present study, it is recommended that N deficiency should be ideally determined according to the threshold of Flav at 1.3 Dualex unit and SPAD value at 38 SPAD unit in the middle growth stage (stem elongation stage, booting stage), [31] but additional field trials are needed for further verification and validation.

## 5. Conclusions

In this study, the effects of Dualex meter and SPAD meter on N nutrition diagnosis in rice were analyzed and evaluated through the interaction of N fertilizer and rice cultivar over more than two years. The results showed that Dualex was effective in estimating Chl content and could overcome the saturation problem that the SPAD meter experiences under conditions of high chlorophyll content. At the same time, Dualex can also effectively estimate the content of flavonoids so as to conduct an early diagnosis of N deficiency in rice, which also has the potential to make predictions earlier than the SPAD value. After analyzing the relationship between different indices and monitoring values, it was observed that the overall monitoring effect of Dualex was better than SPAD. Further analysis of the peak stages of N requirement in rice (early panicle initiation stage, booting stage) helped produce a stable correlation between the Flav value and N deficiency in rice, and helped determine the threshold of recommended N application in rice at Flav value = 1.3 Dualex unit or SPAD value = 38 SPAD units. Overall, it is recommended as a result of the current study that when the measured Flav value > 1.3 Dualex unit or measured SPAD value < 38 SPAD unit, the N status was deficient and more additional fertilizer is necessary in rice.

## Figures and Tables

**Table 1:** Basic infor.mation of N fertilizer rates used in the experiments.

Number of Experiment	Location	Transplanting/Harvest Date	Cultivar	Treatment (kg·hm^−2^)	Sampling Date	Soil Classification
					Day/Month	
Experiment.1 2015	SiHong 118.26° E, 33.37° N	14 June	WYJ-24, NJ-4, LJ-7	N0 = 0	23 July, 30 July	Lime concretion blacks soil
		25 October		N2 = 120	6 August, 13 August	Total N = 1.28 g kg^−1^
				N3 = 240	19 August, 24 August	Olsen P = 27.6 mg kg^−1^
				N5 = 360	12 September	Available K = 75.2 mg kg^−1^
Experiment.2 2015	Huai’An 118.89° E, 33.59° N	22 June	WYJ-24, NJ-4, LJ-7	N0 = 0	25 July, 1 August	Yellow-brown soil
		1 November		N2 = 120	8 August, 15 August	Total N = 1.35 g kg^−1^
				N3 = 240	21 August, 28 August	Olsen P = 32 mg kg^−1^
				N5 = 360	10 September	Available K = 85.3 mg kg^−1^
Experiment.3 2016	SiHong 118.26° E, 33.37° N	25 June	WYJ-24, NJ-4, LJ-7	N0 = 0	22 July, 4 August	Lime concretion blacks soil
		26 October		N2 = 120	15 August, 22 August	Total N = 1.28 g kg^−1^
				N3 = 240	28 August, 10 September	Olsen P = 27.6 mg kg^−1^
				N5 = 360		Available K = 75.2 mg kg^−1^
Experiment.4 2016	RuGao 120.76° E, 32.27° N	18 June	WYJ-24, NJ-4	N0 = 0	16 July, 25 July	Loam soil
		22 October		N1 = 100	2 August, 11 August	Total N = 1.66 g kg^−1^
				N4 = 250	21 August, 26 August	Olsen P = 13.6 mg kg^−1^
				N6 = 400	21 September	Available K = 92.6 mg kg^−1^

Note: “WYJ-24” is “Wunyunjing-24”, “NJ-4” is “Ningjing-4”, “LJ-7” is “Lianjing-7”; “N0–6” means the different nitrogen fertilizer rates.

**Table 2:** The relationship between Dualex values, SPAD readings, and the different N indicators.

N Indicator	Meters’ Indicator	Growth Stage						
		Tillering	Stem Elongation	Panicle Initiation	Booting	Heading	Flowering	Total
LNC	FLAV	0.52 **	0.77 **	0.83 **	0.75 **	0.71 **	0.62 **	0.79 **
	Chl	0.49 **	0.67 **	0.80 **	0.72 **	0.64 **	0.49 *	0.73 **
	NBI	0.47 **	0.69 **	0.72 **	0.67 **	0.67 **	0.45 *	0.75 **
	SPAD	0.35 **	0.76 **	0.74 **	0.63 *	0.73 **	0.61 **	0.70 **
PNC	FLAV	0.59 **	0.74 **	0.76 **	0.74 **	0.70 **	0.56 **	0.69 **
	Chl	0.53 **	0.61 **	0.74 **	0.71 **	0.70 **	0.43 *	0.52 **
	NBI	0.55 **	0.62 **	0.73 **	0.77 **	0.72 **	0.54 **	0.68 **
	SPAD	0.51 **	0.73 **	0.72 **	0.69 **	0.63 **	0.48 *	0.60 **
NNI	FLAV	0.68 **	0.73 **	0.79 **	0.82 **	0.79 **	0.72 **	0.73 **
	Chl	0.66 **	0.68 **	0.79 **	0.76 **	0.68 **	0.72 **	0.66 **
	NBI	0.63 **	0.58 **	0.84 **	0.82 **	0.73 **	0.56 **	0.69 **
	SPAD	0.58 **	0.62 **	0.79 **	0.72 **	0.71 **	0.58 **	0.67 **
NSI	FLAV	0.53 **	0.76 **	0.84 **	0.78 **	0.72 **	0.66 **	0.78 **
	Chl	0.60 **	0.62 **	0.76 **	0.68 **	0.65 **	0.34 *	0.70 **
	NBI	0.57 *	0.61 **	0.67 **	0.84 **	0.64 **	0.42 *	0.74 **
	SPAD	0.45 **	0.66 **	0.76 **	0.67 *	0.70 **	0.64 **	0.65 **
AND	FLAV	0.72 **	0.73 **	0.73 **	0.71 **	0.66 **	0.72 **	0.71 **
	Chl	0.65 **	0.64 **	0.55 **	0.61 **	0.61 **	0.66 *	0.63 **
	NBI	0.68 **	0.71 **	0.63 **	0.62 **	0.58 **	0.72 *	0.69 **
	SPAD	0.55 **	0.64 **	0.65 **	0.68 *	0.62 **	0.68 **	0.65 **
Grain yield	FLAV	0.58 **	0.53 **	0.56 **	0.48 *	0.64 **	0.63 **	0.49 *
	Chl	0.56 **	0.52 **	0.53 **	0.12	0.52 **	0.62 **	0.41 *
	NBI	0.58 **	0.50 **	0.48 *	0.30 *	0.49 *	0.61 **	0.46 *
	SPAD	0.49 **	0.61 **	0.44 *	0.49 *	0.53 **	0.64 **	0.47 *

Note: * Indicating significant difference at 0.05 probability level; ** indicating significant difference at 0.01 probability level.

## Abbreviations

RMSE	root mean square error
RRMSE	relative RMSE
AND	accumulated nitrogen deficit
NBI	nitrogen balance index
Flav	flavonoid
Chl	chlorophyll
TI	tillering
SE	stem elongation
PI	panicle initiation
BT	booting
HD	heading
GF	grain filling
NNI	nitrogen nutrition index
NSI	nitrogen sufficiency index
RAGDD	calculation of Relative Accumulated Growing Degree Days
